# Computer simulation of syringomyelia in dogs

**DOI:** 10.1186/s12917-018-1410-7

**Published:** 2018-03-09

**Authors:** Srdjan Cirovic, Robert Lloyd, Jelena Jovanovik, Holger A. Volk, Clare Rusbridge

**Affiliations:** 1 The Centre for Biomedical Engineering, University of Surrey, Guildford, GU2 7XH UK; 2 The School of Veterinary Medicine, University of Surrey, Guildford, GU2 7AD UK; 3 Fitzpatrick Referrals, Eashing, Halfway Lane, Goadalming, GU7 2QQ UK; 4 Clinical Science and Services, Royal Veterinary College, Hatfield, AL9 7TA UK; 5 Neuroscience Research Australia and Prince of Wales Clinical School, University of New South Wales, Sydney, NSW 2031 Australia

**Keywords:** Cavalier king Charles spaniels, Mathematical modelling, Chiari malformation, Pathophysiology, Syrinx

## Abstract

**Background:**

Syringomyelia is a pathological condition in which fluid-filled cavities (syringes) form and expand in the spinal cord. Syringomyelia is often linked with obstruction of the craniocervical junction and a Chiari malformation, which is similar in both humans and animals. Some brachycephalic toy breed dogs such as Cavalier King Charles Spaniels (CKCS) are particularly predisposed. The exact mechanism of the formation of syringomyelia is undetermined and consequently with the lack of clinical explanation, engineers and mathematicians have resorted to computer models to identify possible physical mechanisms that can lead to syringes. We developed a computer model of the spinal cavity of a CKCS suffering from a large syrinx. The model was excited at the cranial end to simulate the movement of the cerebrospinal fluid (CSF) and the spinal cord due to the shift of blood volume in the cranium related to the cardiac cycle. To simulate the normal condition, the movement was prescribed to the CSF. To simulate the pathological condition, the movement of CSF was blocked.

**Results:**

For normal conditions the pressure in the SAS was approximately 400 Pa and the same applied to all stress components in the spinal cord. The stress was uniformly distributed along the length of the spinal cord. When the blockage between the cranial and spinal CSF spaces forced the cord to move with the cardiac cycle, shear and axial normal stresses in the cord increased significantly. The sites where the elevated stress was most pronounced coincided with the axial locations where the syringes typically form, but they were at the perimeter rather than in the central portion of the cord. This elevated stress originated from the bending of the cord at the locations where its curvature was high.

**Conclusions:**

The results suggest that it is possible that repetitive stressing of the spinal cord caused by its exaggerated movement could be a cause for the formation of initial syringes. Further consideration of factors such as cord tethering and the difference in mechanical properties of white and grey matter is needed to fully explore this possibility.

**Electronic supplementary material:**

The online version of this article (10.1186/s12917-018-1410-7) contains supplementary material, which is available to authorized users.

## Background

Syringomyelia is a severe progressive pathological condition characterised by the presence of abnormal fluid-filled cavities (singular syrinx and plural syringes) within the spinal cord [1–8]. Clinical signs of syringomyelia include pain, loss of sensation, and motor dysfunction [2]. Syringomyelia affects both humans and animals (mainly dogs and cats) [9–11]. In humans, syringomyelia has a low prevalence of about one in 10,000 [3, 4]. In contrast, in some toy breed of dogs such as Cavalier King Charles Spaniels (CKCS), King Charles spaniels, Griffon Bruxellois, Maltese, Chihuahuas, and Pomeranians the prevalence is high. In the CKCS up to 70% of the total population is affected by the condition although in many dogs it may be asymptomatic [12]; in the Griffon Bruxellois, 50% of the American population was affected with again many dogs being asymptomatic [11].

Syringomyelia is associated with the obstruction of CSF channels and the subarachnoid space (SAS), particularly if the obstruction is at the foramen magnum [4]. The most common cause of foramen magnum obstruction in humans is Chiari malformation and in animals the analogous condition, Chiari-like malformation, to which brachycephalic toy breed dogs such as Cavalier King Charles Spaniels (CKCS) are particularly predisposed [13]. A common feature of Chiari-like malformation in several toy breeds is occipital bone hypoplasia and reduced caudal fossa volume associated with a compensatory increase in height of the cranial fossa [14]. Traits that increase the risk of syringomyelia in CKCS include brachycephaly with rostral displacement of the atlas and axis, acute angulation between the sphenoid and basioccipital bone, reduced occipital crest and increased cervical flexure and odontoid (dens) angulation [14–17]. Chiari-like malformation is analogous to Chiari type 1 and 0 malformation in humans which is also associated with syringomyelia [18].

The pathogenesis of syringomyelia is still unknown and this ultimately compromises the effectiveness of clinical (surgical) management of syringomyelia [2, 4, 8, 19]. It is widely believed that variation to the normal flow pathways of the cerebrospinal fluid (CSF) plays the main role in the formation and expansion of syringes [20]. CSF constantly moves in a pulsatile manner between the cranial and spinal SAS via the foramen magnum to compensate for transient shifts in the cranial blood volume associated with the arterial pulse [21, 22]. In Chiari malformation, the obstruction of the foramen magnum compromises the normal communication between the cranial and spinal CSF compartments [3, 23]. Similarly, stenoses in the SAS due to traumatic injury obstruct the flow of CSF [4]. In-vivo and in-vitro experiments [24–27] indicate that such blockages can result in pressure dissociation in the SAS and in excessive motion of the spinal cord [28]. Typically, syringes form caudally from a blockage. In dogs most affected with syringomyelia, the emergence and progression of cavities has a breed-related pattern. Thus, for example in Cavalier King Charles Spaniels the initial cavities usually first emerge in the cranial-cervical and thoracic-spinal cord segments (C1-C4 and T1-T4, respectively) [29]. The twelfth thoracic-second lumbar (T12-L2) segment is also a common site where syringes may form [29]. By comparison, in some breeds such as Griffon Bruxellois the cavities are often widest in the cervicothoracic junction and cranial thoracic region (unpublished data). In humans the analogous condition Chiari-I malformation is more likely to be associated with a cervical syringe [30].

Early theories on the origins of syringomyelia speculated that syringes form because bulk CSF flow is redirected to the central canal of the spinal cord which is connected with the ventricular system of the brain [1, 25]. However, these theories were abandoned since they could not account for non-caninular syringes (not continuous with the central canal) or syringes emerging in the absence of a patent central canal [20]. Current theories focus mainly on the accumulation of fluid into the spinal cord parenchyma [31–37] and on excessive stressing of the spinal cord tissue [38–40]. Most of the current theories are derived from in-vitro and computer models that can simulate propagation of CSF pulse waves in the spine under various scenarios [31–35, 38–50]. These models can capture the interaction between elastic structures (spinal cord and dura) and the CSF as well as predict spatial and temporal variation of parameters such as CSF pressure, CSF velocity, and stress in the spinal cord. Cirovic et al. [41, 49], and Bertram et al. [39, 51] used mathematical/computer models to identify four pulse modes in the spinal cord. Three of the four modes are involved in the movement of the CSF and have speeds of the order of several metres per second. More recently Støverud et al. predicted pulse speeds between 2 and 5 m/s using an anatomically realistic computer model of poroelastic spinal cord [52]. The findings from the computer modelling studies are consistent with the pulse speed data obtained from in-vivo human and animal experiments [24, 53, 54]. Elliot [40] further examined the pulse modes and concluded that stenoses in the SAS lead to elevated stress in the cord which is released with the emergence of a syrinx (branding this “a homeostatic mechanism for syringomyelia”). Bertram developed a fluid-structure interaction computer model of the human spinal cavity that included the cord with a syrinx, the SAS fluid compartment, and the elastic dura [38, 39, 45]. The model geometry was idealised to be axisymmetric neglecting the spinal curvature. He concluded that arachnoiditis can lead to excessive stressing of the cord, and that the sloshing of the fluid in an existing syrinx can induce stresses at the caudal end of the syrinx. Recently, Bertram and Heil [32, 35] extended Bertram’s original model to examine the case of a stenosis overlaying a large syrinx for a poro-elastic spinal cord. They concluded that this situation results in a “diastolic valve” that may pump small amounts of fluid from the SAS to the syrinx with each cardiac cycle. While these results are promising, they mainly apply to the enlargement of an existing syrinx or a “presyrinx” (an initial state of increased fluid in the spinal cord [55]) rather than with its emergence. None of the models convincingly explain why syringes emerge at specific anatomical locations even in the absence of arachnoiditis or an initial small “pre-syrinx”.

We hypothesize that preferential locations for the emergence of syringes may be related to the curvature of the spine. This cannot be examined with existing computer models of syringomyelia since they all approximate the spinal cord as a straight cylindrical structure. In order to investigate this possibility, we constructed a three-dimensional computer model of the canine spine. The CKCS spine was chosen for the study due to a high prevalence of syringomyelia in this breed and because of a highly regular pattern in the development of cavities in the spinal cord.

## Methods

The dynamics of the canine spinal cord was investigated using a finite element model of a canine spinal cavity. The finite element method is an approximate method for solving engineering problems involving complex geometries and material properties that cannot be solved analytically. In this method, the domain of interest is “discretized” by breaking it into a large number of small geometric components called “elements”. For each element in the model, simplifying approximations are applied to the equations governing the mechanical behaviour of the system examined. The resulting set of equations involving all the elements is solved using computers to yield quantities such as displacement, speed, strain and stress in each element.

### The model

**Fig. 1:**
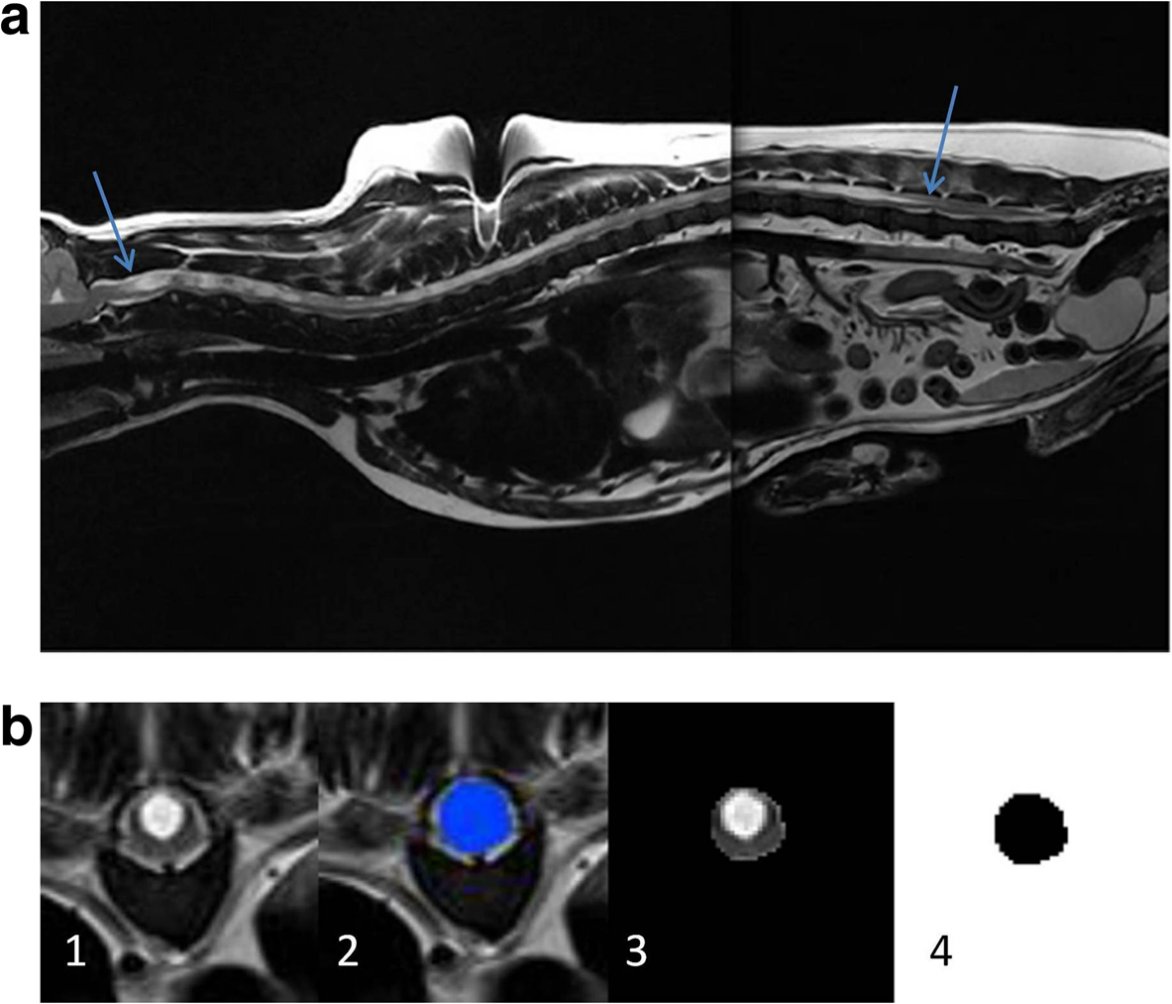
**a** T2-weighted mid-sagittal MRI of a CKCS suffering from a large syrinx (syrinx ends indicated with arrows). **b** The geometry reconstruction process. 68 transverse plane scans were obtained (1). Each anatomical layer was segmented to create a mask (2). The mask was filtered out (3) and then binarised (4) to create a pure black and white image

The computational model was developed from a CKCS with a Chiari-like malformation and fully developed syrinx (C2-L3; Fig. 1a). The geometry of the spinal canal was extracted from T2-weighted transverse anatomical magnetic resonance imaging (MRI) scans. The imaging was obtained as part of the necessary diagnostic work and consent was obtained from the owner. Due to the size of the subject, two series of transverse scans were compiled to cover the full length of the spine (C1 – S1) at 5 mm intervals with a 10% interslice gap. During the MRI scan the dog was placed in dorsal recumbency with its pelvic limbs extended caudally on the scanning couch, with the spine being as straight as possible to avoid any external distortions of the images. The following T2-weighted imaging parameters were applied: Time to Repetition (TR) 3200, Echo Time (TE) 102, Field of View (FOV) 150, matrix resolution 384 × 320, and averages 5, using combination of spine table integrated coil and torso array coil for maximum signal collection.

**Fig. 2:**
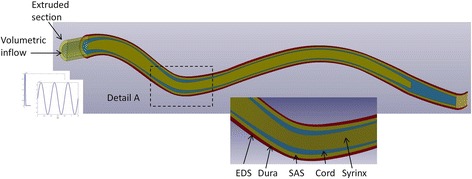
The finite element model of the CKCS suffering from a large syrinx. The anatomical layers are indicated in Detail A. The transparent cranial segment was added to the original geometry and the inputs were applied at its cranial end

In order to reduce the complexity of the model and create a high quality mesh, the components of the spinal canal were assumed to be circular and concentric. Previous studies have demonstrated that the CSF pressures are relatively unaffected by the concentricity and shape of the anatomic layers [56, 57]. The geometry of the spinal canal (C2 - S1) was manually segmented using the Simpleware software package (Simpleware Ltd., Exeter, UK) generating two-dimensional (2D) transverse masks for the following components of the spinal canal; the epidural space (EDS), the subarachnoid space, the spinal cord and the syrinx cavity (Fig. 1b). The 2D masks were exported into ImageJ, an open source image processing platform [58], to calculate the cross-sectional areas and the corresponding radii of each component. The centroids of the spinal cord masks were assumed to be the centres for all components. The reconstructed geometric data is summarised in Table 1 and in the Additional file 1.xlsx. A volumetric model was generated in ANSYS (ANSYS Inc., Pittsburgh USA) by connecting the transverse sections with splines. It was found that it is sufficient to use only 13 sections to construct a smooth three-dimensional geometry that conforms well with the reconstructed 68 transverse sections (see Table 1). The spine was assumed to be symmetric about the mid-sagittal plane and medio-lateral motion was neglected. The cranial end of the model was extended by 15 mm (transparent section in Fig. 2). This was done to remove spurious stress concentrations which would form in the cranial end of spine if the model was loaded directly at the C2 section of the model. Hexagonal “brick” elements of 0.5 mm overall size were used to discretize the model. The element size was chosen so that each layer has at least two elements across the thickness. The finite element model comprised of 903,232 elements in total. Figure 2 detail A shows all the layers in the final model, with the finite element mesh. The dura mater was modelled as a layer of shell elements with 1 mm thickness between the SAS and the EDS.

**Table 1:** Geometric parameters of the model

x [mm]	y[mm]	R_syrinx [mm]	R_cord [mm]	R_SAS [mm]	R_EDS [mm]
0	0	2.2	4.5	6.4	7.2
20	3.4	3.7	4.6	5.6	6.3
45	−7.9	4.5	5.1	6.3	7.1
75	−22.8	2.5	4.3	5.6	6.2
95	−24.2	2.6	3.8	5.1	6.0
125	−20.0	3.0	3.6	4.5	5.4
170	−9.3	3.1	3.5	4.6	5.5
205	−5.7	2.7	3.3	4.3	5.1
245	−12.0	3.0	3.7	4.9	5.5
275	−22.3	2.4	3.9	5.3	6.2
285	−25.7	1.8	4.1	5.2	6.2
325	−35.2	–	2.5	4.0	5.0
335	−34.5	–	–	3.8	4.3

Coordinates of the centroids and radii of the syrinx, cord, SAS, and EDS used in the model. The x coordinate is in the cranial-to-caudal direction. The y coordinate is in the ventral-to-dorsal direction. The raw data for syrinx, cord, and SAS radii and centroids are given in the Additional file 1

The mechanical properties for the materials of the layers comprising the model were taken from the published literature [20]; the cord, dura, and the epidural fat were represented as linear elastic solids, and the CSF was represented as an inviscid fluid with the compressibility equal to that of water (Table 2). Treating CSF as inviscid in the first approximation is justified since the pulsatile flow of the CSF in the spine is dominated by inertia rather than viscous effects [56]. From a practical point of view, inviscid approximation was useful since it allowed for the simultaneous solution of solid and fluid equations using a Lagrangian approach. The model was constrained from moving at the outer edge of the EDS to account for the constraint imposed by the vertebrae. Constraint to movement was also imposed to the caudal ends of the spinal cord and the SAS.

**Table 2:** Material properties

Spinal cord	E = 62.5 kPa, ν = 0.49, ρ = 1000 kg/m^3^
Dura	E = 1.25 MPa, ν = 0.4, ρ = 1000 kg/m^3^
EDS fat tissue	E = 1 kPa, ν = 0.25, ρ = 900 kg/m^3^
CSF and the fluid in the syrinx	K = 2.2 GPa, G = 0, ρ = 1000 kg/m^3^

Material properties of the anatomical layers. E = Young’s modulus, ν = Poisson’s ratio, K = bulk modulus, G = shear modulus, ρ = density

### Simulations

An input with amplitude and frequency representative of CSF movement generated by the arterial pulse in the cranium were used to excite the model. This was done by imposing a sinusoidal volumetric flow with a period of 0.4 s (150 beats per minute as the upper limit of heart rate for CKCS [59]) and an amplitude of 0.35 cm^3^/s. This resulted in a peak CSF speed of about 0.02 m/s which is within a physiologically plausible range [28, 53, 56, 60–62]. The volumetric input was applied at the cranial end of the extruded section of the model. Three extreme hypothetical situations were considered by varying the boundary conditions at the cranial end of the extruded section and by excluding or including the syrinx:

1. Cord without syrinx; cranial end of the cord constrained from movement and the CSF allowed to flow freely at the cranial end (representing the normal condition).
2. Cord without syrinx; cranial end of the cord allowed to move freely and the CSF blocked at the cranial end (representing Chiari with the syrinx still not formed).
3. Cord with syrinx; cranial end of the cord allowed to move freely and the CSF blocked at the cranial end (representing Chiari with the syrinx formed).

Prior to applying the above listed scenarios, a preliminary simulation was run in which the model was excited with a short transient pulse of 0.01 s duration and 0.35 cm^3^/s volumetric flow amplitude. This was done to test if the model can predict the pulse modes reported in the published theoretical and computational studies. All simulations were performed using the LS-DYNA finite element package for dynamic simulations (LS-DYNA Version 971, Livermore Software Technology Corp., Livermore, CA).

## Results

**Fig. 3:**
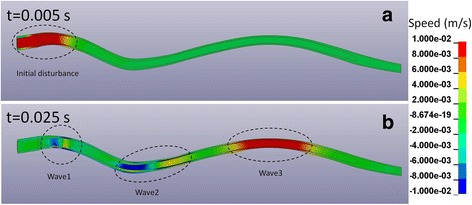
Axial (cranial-to-caudal) speed of tissue movement in the spinal column due to a short transient cranial excitation. **a** At *t* = 0.005 s from the onset of excitation an initial disturbance with the amplitude of 0.01 m/s (1 cm/s) formed at the cranial end. **b** At *t* = 0.025 s the initial disturbance split into three waves which travelled at speeds of 1.5 m/s (wave 1), 7 m/s (wave 2), and 11 m/s (wave 3). The waves generated tissue movement in the range of ±0.01 m/s

Figure 3 shows the distribution of the axial (cranial-to-caudal) component of speed for a short pulse excitation. Only the syrinx, cord, and SAS layers are shown since the movement in the EDS was very small. At 5 ms after the excitation, there was a single disturbance at the cranial end of the model. At 25 ms that disturbance split into three waves that moved at different speeds and covered different distances. For the fastest wave modes, all three layers moved in the same (caudal) direction. For the second fastest mode, the fluids in the syrinx and SAS moved in the opposite direction from the spinal cord. For the slowest mode, the syrinx and the spinal cord moved in the opposite direction from the CSF in the SAS. From the distance covered and the time elapsed, the speeds of the three modes can be estimated to be approximately 1.5 m/s, 7 m/s, and 11 m/s. The speed of movement of the tissues in the anatomical layers of the spine generated by the pulse modes is of the order of 10^− 2^ m/s (see Fig. 3).

The “cardiac” excitation of the model generated an oscillatory motion of the SAS and/or spinal cord at the cranial end of the model. For the “normal condition” the peak speed of the CSF at the cranial end of the SAS was approximately 0.02 m/s. The amplitude of the speed in the SAS diminished in a linear way from the cranial to the caudal end. For the “Chiari” conditions, the peak speed of the cord at the cranial end was just below 0.01 m/s and the same linear tendency of amplitude decrease towards the caudal end was observed. In all three cases considered, the excitation resulted in harmonic pressure fluctuations in the SAS with a peak amplitude of approximately 400 Pa (about 3 mmHg). The pressure amplitude was approximately constant along the length of the spine; the pressure gradient between the cranial and the caudal ends did not exceed 0.5 mmHg over the cardiac cycle.

**Fig. 4:**
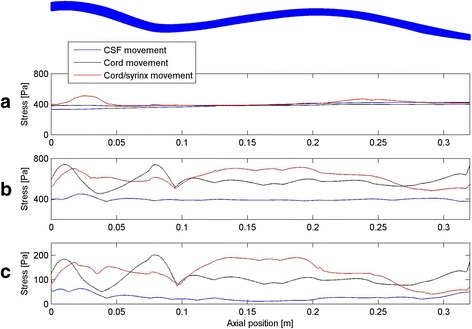
The distribution of maximal stress in the axial (cranial-to-caudal) direction along the spinal cord. The axial coordinate is measured from C2; the shape of the spinal cord is shown at the top for reference. **a** Radial normal stress. **b** Axial normal stress. **c** Shear stress

**Fig. 5:**
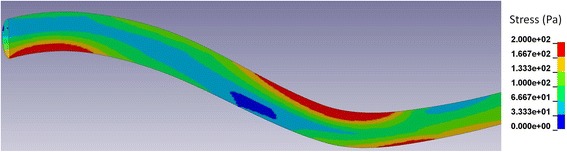
The distribution of peak shear stress in the cervical and cranial thoracic part of the spinal cord (Chiari without a syrinx case). The regions in red are the locations of the highest shear stress

Figure 4 shows the distribution of stress in the cord along the axis of the spine for the three inputs. The displayed results were obtained in the following way: for each element comprising the discretized spinal cord, a stress-versus-time curve was obtained and the peak value of stress was determined. Next, the cord was divided into segments of one millimetre width in the cranial-to-caudal direction and the maximal value of peak stress recorded in the elements within each segment was determined. Thus, each point in a curve represents the maximal value of stress within a 1 mm thick slice of the cord over the whole simulated event. Please note that the normal stresses in the cord were entirely negative (compressive) and that the values shown represent the absolute maximal values. The radial stress perpendicular to the sagittal plane is shown in Fig. 4a. The stress was fairly uniform along the length of the cord for all three cases considered and approximately equal to the pressure in the SAS (400 Pa). The same was found for the radial stress in the sagittal plane (not shown in the figure). The radial stress was thus a reflection of the pressure in the SAS. For the normal case, the axial normal stress (cranial-to-caudal direction) showed little variation along the length of the spine and had the same amplitude as the radial stress. In contrast, for the Chiari case without a syrinx, the axial normal stress had two prominent peaks of just under 800 Pa, which were located at the level of C2 and C3 vertebrae and cervico-thoracic junction. Elsewhere, the stress values were about 200 Pa higher than for the normal condition. With the syrinx introduced, the stress levels remained elevated, the peaks were less distinct and there was a further stress increase in the lower thoracic portion of the spinal cord. This was also observed for the shear stress, as can be seen in Fig. 5c. For the normal condition, the shear stress was very low, an order of magnitude lower than the normal stress. For Chiari, the shear stress was considerably elevated with two peaks of approximately 200 Pa at the level of C2 and C3 vertebrae and cervico-thoracic junction. To get a more complete understanding as to the location of maximal stresses, we must look at the three-dimensional distribution. Figure 5 shows the distribution of the peak shear stress in the cranial portion of the spinal cord for the case of Chiari without a syrinx. The results displayed were obtained by assigning to each element the peak value of the shear stress recorded in that element during the simulated event. It can be seen that regions with the highest level of stress were on the ventral side of the cranial-cervical cord and at the dorsal side of the caudal-cervical/cranial thoracic spine. The same type of result was obtained for the normal axial stress. This suggests that regions of localised high stress are due to the bending deformation of the spinal cord.

## Discussion

The results for the short transient excitation show that the model can successfully predict the wave modes of the CSF pulse propagation reported in the open literature. The speeds of the three modes estimated from the results, as well as the associated movement of the anatomical layers are consistent with the theoretical models presented by Cirovic [41, 49]. They are also within the physiologically plausible range determined from in-vivo experiments on humans and animals [53, 54]. As the previous theoretical and computational studies neglected the curvature of the spine, it can be concluded that the curvature has a negligible effect on the propagation of the CSF pulse. The spinal curvature also had little effect on the pressure generated in the SAS; for all three cases considered, the pressure amplitude in the SAS was approximately 400 Pa, and it was uniform along the length of the spine. The predicted small spatial pressure gradient is consistent with findings of other published studies [63]. In other words, the spinal cavity behaved as a lumped compartment whose compliance was chiefly determined by the elasticity of the dural sac. This is because the time scales of the cardiac input are larger than the time needed for the CSF pulse to traverse the entire spinal cord. For all cases considered all the stress components in the cord were of the same order of magnitude as the pressure in the SAS (several hundreds of Pa). For the normal case where the cranial end of the cord was fixed, all the components of the normal stress were compressive and effectively equal to the pressure in the SAS. In other words, the pressure in the SAS generated an equal “hydrostatic pressure” (average normal stress) in the spinal cord. When the CSF was blocked and the cord was allowed to move, the radial normal stress did not change, but the axial (cranio-caudal) stress increased. We attribute this result to the fact that the axial movement of the cord resulted in bending in the sagittal plane. Bending deformation generates normal axial stresses that are largest at the perimeter of the section, and this is consistent with the predictions of the model. It should be noted that the maximal displacement of the cord in the caudal direction was small; about 1 mm in total. The axial normal stress was particularly prominent at the locations where the spinal cord had large curvature and the bending effect was pronounced. The same case was for the shear stress which is also generated by bending. Furthermore, these locations are also the locations where the cavities usually first start forming in the CKCS affected with syringomyelia. When a large syrinx was present, stress concentration moved to the lower thoracic region which is the next most likely site of syrinx formation [29]. This is an interesting result, and to our knowledge this is the first case where computer simulation predicts stress concentration at specific regions of the spinal cord in absence of stenosis and arachnoiditis. Although the stress magnitude at the most affected regions increased by as much as 100% from the normal values, the overall stress levels were generally quite low, and should not be able to cause an immediate damage to the spinal cord tissue. However, it is still feasible that exaggerated stressing repeated with each cardiac pulse does cause pathological changes to the spinal cord tissue over a long period of time. Actually, this is consistent with the fact that syringes take months or years to form [2, 8]. While the axial location of the increased cord stressing is coincident with the sites where the syringes tend to form, the model predicted that the stress was concentrated at the perimeter of the spinal cord rather than at the central portion. This is not in agreement with the fact that the cavities are usually located more centrally. Therefore, as it stands now, the results of this study cannot fully support the assumption that stressing of the cord near the maximum curvature locations may initiate the formation of syringes via direct mechanical damage. However, it should be considered that the model presented in this study does not include numerous factors that could modify the current result. Such factors include non-concentric realistic geometry of the anatomical layers, different material properties of the white and grey matter of the spinal cord, and the effect of denticulate ligaments and tethering in general. It is also possible that the syrinx initiation mechanism is less direct; it has been shown that the damage of microstructure at the surface of the cord may disrupt the blood-spinal cord barrier and affect fluid content of the spinal cord [37, 64, 65], thus leading to fluid accumulation.

Similar to other computer models, the model presented in this paper is based on a number of idealisations. Furthermore, the material properties of the spinal compartments are still not well characterised. We opted for choosing the material properties of the dura and the spinal cord that are most commonly used in the current (human) models of the spinal cavity [32, 35, 38, 39, 45]. Prior to making this choice, the model had been tested with the full range of spinal cord and dura material properties reported in the literature as summarised by Elliot et al. [20]. The tests showed that the dura properties listed in Table 2 lead to the most realistic results in terms of SAS pressure magnitude. When a lower stiffness was assigned to the spinal cord tissue, this resulted in proportionately lower stresses in the cord. However, the general trends remained the same; there was a significant increase in the axial and shear stresses at the upper cervical and thoracic spinal cord for the Chiari condition. This suggests that the conclusions for the current model are largely independent of the material constants, as long as they are within a plausible range. On the other hand, this also means that all the quantitative values of stress reported in this paper should not be taken literally, but rather in reference to the set of material properties used in the current model.

Virtually all computer models of syringomyelia end at the cranial end of the cervical spine or at best extend into the cranio-cervical junction. In this model, an ad hoc extruded cranial segment was added to avoid spurious stress concentrations at the cranial end of the cord. Effectively, this problem arises from the fact that the brain and the cranium are not included in the model. As Chiari deformation results in the brain being “crammed” in the cranium, it is possible that the lower portion of the brain and perhaps cranial section of the cervical spinal cord are under permanent stressing. Therefore, ultimately, a more complete model of syringomyelia should include both the cranial and spinal compartments.

## Conclusions

A computer model was used to investigate the effect of excess movement of the spinal cord in dogs suffering from Chiari-like malformations with the goal of identifying possible factors that could promote the emergence of fluid-filled cavities in the spinal cord. It was found that small movements of the spinal cord associated with a cardiac pulse can result in a significant increase in shear and axial normal stresses and that this effect is most pronounced in the cervical and upper thoracic regions where the spinal curvature is high. The regions of high stress as predicted by the model are at the locations where syringes first form, but they are positioned near the perimeter, rather than at the central portion of the cord which is where the cavities usually are. We believe that the latter may be due to simplifying assumptions inherent to the model, and that this mechanism should be further investigated.

## Additional file

Additional file 1: Geometric parameters of the model.ᅟ(XLSX 18 kb)
